# Modelling of the Guillotine Cutting Process by Means of a Symmetrical Blade with the Defined Geometry

**DOI:** 10.3390/ma13235404

**Published:** 2020-11-27

**Authors:** Jarosław Kaczmarczyk

**Affiliations:** Department of Theoretical and Applied Mechanics, Silesian University of Technology, 18A Konarskiego Street, 44-100 Gliwice, Poland; jaroslaw.kaczmarczyk@polsl.pl

**Keywords:** ultra-thin steel sheet, bundle of metal sheets, finite element method, scanning electron microscopy, nonlinear analysis, metal fracture, plastic and brittle cracking, plastic-brittle transition

## Abstract

This paper modelled the cutting process of a bundle consisted of ultra-thin cold-rolled steel sheets using a guillotine. The geometry of a cutting tool with given dimensions was assumed. A bundle of sheets being cut was modelled as deformable, the cutting tool was rigid, and the finite element method along with computer system LS-DYNA was employed. Numerical simulations of the complex state of stress and of the corresponding complex state of strain were carried out. Cutting processes belong to fast changing physical phenomena, and therefore, highly nonlinear dynamical algorithms were applied in order to solve this particular problem. Experimental investigations were also conducted by means of the scanning electron microscopy. It was found that the fracture region consisted of two distinct zones: brittle and ductile separated from each other by the interfacial transition. Morphological features of the brittle, ductile, and the transition regions were identified. The ductile and brittle zones were separated at the depth of ca. 1/5 thickness of the cut steel sheet. Finally, the numerical results obtained by usage of the finite element method as well as experimental ones in the form of microscopic images were compared, showing quite good agreement.

## 1. Introduction

The cutting process is very often used in everyday life as well as in many branches of industry—for example, in the printing industry as matrices for printing books, newspapers, magazines, etc., in the production of metal cans as well as foil for food storage and in automotive industry for cutting out car bodies. There are well-known different kinds of cutting techniques [1,2], for example: water jet, laser beam, plasma, scissors, lathe, guillotine, slitters, etc. The applied technique depends on each user’s needs. It should be highlighted that the designing of the cutting process is in contradiction of very common safe designing of e.g., machines [3,4,5,6], various mechanical constructions with repeating cyclically fatigue strength [7], composite-based materials [8,9], etc. The mentioned safe designing consisted mainly in gaining lower stress or equal to the permissible one [10]. In cutting techniques, the stress should exceed the permissible one; otherwise, the cutting could not be possible [11,12,13,14,15,16].

The cutting of ultra-thin cold rolled steel sheets is highly important in terms of modern industry, but there are many technological problems encountered during the process, such as too high deflection of the edge bending, too small brittle zone, and a too large plastic zone, which influenced the quality of the sheets being cut.

It is feasible to cut a single metal sheet or a bundle of ultra-thin metal sheets, but it is rather obvious that the cutting of a bundle of sheets is more effective [17,18,19,20,21] than cutting a single sheet [22,23,24]. Another approach is related to the geometry of a cutting tool. A research of the cutting process utilising a non-symmetrical cutting tool has been elaborated by Kaczmarczyk et al. [17,23]. In those papers, the experimental as well as numerical investigations concerning the cutting of one [23] and two separated sheets [17] made of steel C75S have been analysed and thoroughly discussed. The metal sheet being cut has been modelled as a deformable and the cutting tool has been modelled as a non-symmetrical rigid body.

The cutting process has become more and more popular in-home industry as well as all around the world. The modelling of the cutting process would not be possible without introducing the appropriate physical model concerning the propagation of fracture. In the literature, many different such physical models are proposed [25,26,27,28,29]. Currently, the cutting productivity might be increased by changing the technology of the process passing from cutting a single metal sheet into many sheets arranged in a bundle and being cut at once. Furthermore, the effectiveness of cutting might be raised by increasing the number of metal sheets in a bundle; however, the higher the bundle is (the more metal sheets create the stack), the more problems occur during cutting [17,18,19,20,21]. The most common problems with cutting the metal sheets in a bundle are as follows: edge bending, burrs, vertical scratches in the shape of craters, etc. The experiment shows that the more metal sheets are in a bundle, the higher edge bending is, the more serious random craters as well as the more frequently burrs occur [20]. In order to reduce the early mentioned issues understood as the reduction of the deflection of the edge bending, the random occurrence of scratches minimising the size of craters, as well as the random frequency of craters’ occurrence, classical [30,31], genetic [32,33], or hybrid optimisation methods [34,35,36,37,38] should be applied. The problems seem to be very difficult to solve. Furthermore, it is rather impossible to maximise the height of a bundle of sheets understood as an increase of the number of sheets being cut with a simultaneous reduction of the force required for cutting, reduction of the deflection in edge bending, and a decrease in the frequency of random occurrence of the craters as well as in the size of scratches. However, it is possible to apply a multi-criteria optimisation [32,33,38] instead of a single criterion optimisation with several design variables [30,31]. The multi-criteria optimisation consists of a compromise between the maximum height of a bundle being cut, which entails the maximum possible number of metal plates, the maximum acceptable deflection of the edge bending, the maximum admissible height of craters, and the maximum possible random frequency of vertical craters.

Each time a cutting process is designed to satisfy human requirements, the creator tries to achieve the best solution for the task and therefore conducts optimisation. The optimisation process might be manual, time consuming, and consist of a step-by-step approach to obtain the right set of technological parameters. Many times, the manual industrial approach does not allow for a thorough exploration of the possible solutions in order to find the optimum design variables. In the consumption society, it is imperative to design processes in the most effective way. In real life, identification of the optimum process of the industrial problem is not often possible because of the gravity and large size of the problem as well as the lack of specialised knowledge. In such a situation, the process optimisation is an essential improvement in modern industry.

Many authors address the issue of optimisation in a broad sense using classical, evolutionary, or heuristic methods in minimisation; for example, Tiwari and Chakraborti [38] described a method of optimising the layout of rectangular parts placed on a rectangular sheet to cut out various parts. Two types of cutting problems have been investigated by them in which guillotine cutting has been required mostly for metallic sheets where each cut has been made individually for a single sheet and cuts that can be made using a punch, i.e., for materials such as paper or rubber, where the sheets to be cut can be laid side by side or on top of one another, and a single cut can be made. For the optimisation of the layout of rectangular parts, they used two design objectives involving minimisation of the length of the mother sheet and also the total number of cuts required to obtain all the parts from the mother sheet. They applied a multi-objective genetic algorithm to study both guillotine and non-guillotine cutting cases using a binary representation of the variables.

The novelty of the proposed methods in this paper consists of their application to modelling of the cutting process with different blade geometry. In the previous authors’ articles [17,23], the blade of a cutting tool was non-symmetrical, and that is why the mechanism of cutting was different. Hence, the geometry changes make it possible to reduce the undesirable plastic region in comparison with the size of the plastic zone produced by a non-symmetrical cutting tool. It turned out that the application of a symmetrical cutting tool has positive effects on the performance of the cutting process what means the reduction of the undesired plastic zone (with numerous defects) and simultaneous maximisation of the size of desired brittle zone (without defects).

The main goal of this paper is to present the mechanism of cutting of a bundle comprising metal sheets by means of a symmetrical cutting tool. On the basis of the current paper and earlier author’s work on cutting using a non-symmetrical cutting tool under almost the same cutting conditions [17], one can achieve a significant reduction of the undesired height of a plastic region responsible for many defects and simultaneous increase of a brittle zone, which is strongly desired.

## 2. Materials and Methods

A bundle comprising of ultra-thin cold rolled steel sheets made of C75S (high-strength steel) has been submitted to the cutting process using a symmetrical cutting tool made of high-speed steel (NC10/1.2201). The author’s physical model of the station for carrying out the cutting process and corresponding to it the mathematical one have been elaborated by means of the finite element method [39,40,41,42,43] and computer system LS-DYNA [44] (LSTC, Livermore, CA, USA). The cutting tool as well as the surface of metal sheets being cut have been subjected to observation by means of scanning electron microscopy (SEM) with a cold field emission (FESEM) HITACHI S-4700 (Hitachi, Ltd., Tokyo, Kanto, Japan) equipped with the energy-dispersive X-ray (EDS) NORAN Vantage spectrometer (Noran Co, Vernon, CA, USA).

### 2.1. Mechanical Properties of the Material Being Cut

**Figure 1:**
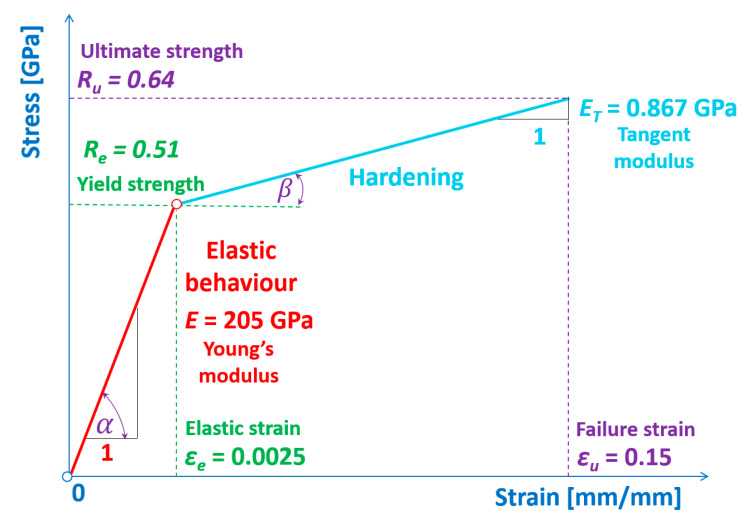
Constitutive bilinear elastic plastic material model of an ultra-thin sheet being cut.

In order to model the process of cutting sheets, the material model has been assumed. The simplest one that takes into account the linear as well as nonlinear behaviour seems to be the so-called the constitutive bilinear elastic plastic material model [45,46], which is depicted in Figure 1. Furthermore, the details of the mechanical properties are juxtaposed in Table 1. The assumed physical model consists of two linear characteristics. The first one describes a linear relationship between the strains and stresses within the Hook’s law, and the slope of this characteristic corresponds to the Young’s modulus (E).

The second characteristic describes the hardening of the material. The slope of this relationship corresponds to the tangent modulus (*E_T_*). The terminal point on this characteristic corresponds to the ultimate strength, which means that if the material reaches this state, it becomes vulnerable to cracking. The tested material cannot sustain higher loads, and therefore, it is the maximum possible load that the material can withstand. Overstepping this particular point means exceeding the permissible forces to which the sample might be subjected without losing the continuity within the whole volume of the material. Above this range, the breakage of the material continuity as well as the further cracking propagation takes place.

In order to simulate the cutting process, it is not enough to assume a constitutive material model, but an additional cracking model is required. For the purpose of this work, the model concerning the cracking process has been assumed and is as follows.

In the beginning of the cutting process, a knife presses the top sheet in a bundle being cut, which generates the forces that should obtain a certain value high enough to initiate the cracking process.

During cutting, the knife modelled as a rigid body interacts mutually with the deformable ultra-thin sheets in such a way that if the calculated Huber–Mises strain acting below the tip of a cutting tool exceeds the ultimate strain corresponding to the material cracking in an uniaxial tensile test; then, in this particular node, the separation takes place—i.e., instead of one single node, the two nodes are created and the additional extra space between them occurs, allowing the cutting tool further penetration into material being cut. Simultaneously, the cutting process develops until the material is separated into two pieces.

In case when the calculated equivalent Huber–Mises strain acting below the tip of a cutting tool in the material being cut is less than the strain corresponding to the ultimate strength, the cracking process is halted; however, it does not mean that the cutting process is finished. The equivalent Huber–Mises stress and corresponding to it equivalent Huber–Mises strain grow until the calculated strain exceeds the ultimate one, and the node separation process described earlier takes place, and the cracking is developing. The defined contact works further, and the cutting process is going forwards; the cutting tool moves downwards until the material is thoroughly separated.

### 2.2. Modelling of the Cutting Process

**Figure 2:**
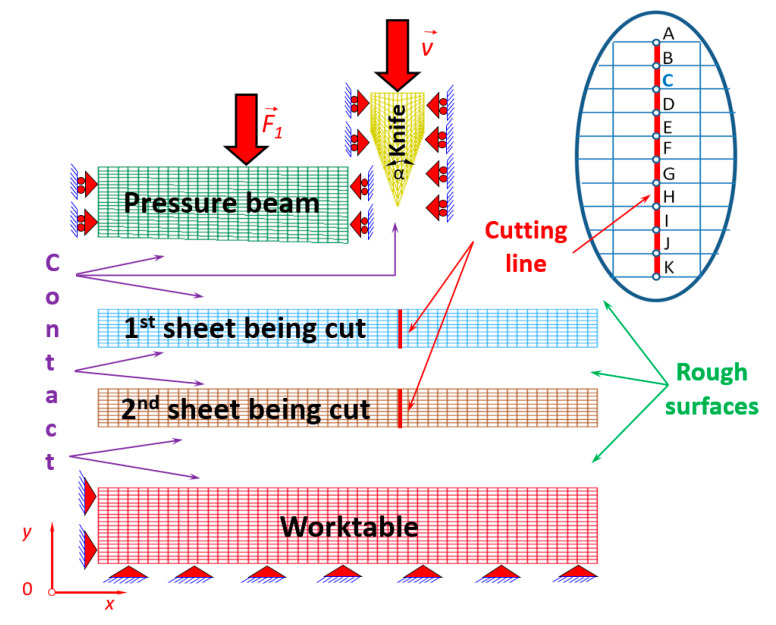
Mesh of the station for carrying out the cutting process with nodes belonging to the cutting line and represented by letters from A to K.

The geometry and corresponding to it a model comprising of two metal sheets in a bundle have been assumed (Figure 2), but it is enough to show the working of the cutting process of sheets bundles. The elaboration of a more sophisticated model consisting of many sheets is connected with a large amount of work concerning the creation of the physical model and the usage of a huge number of the finite elements, introducing the unilateral constrains between sheets (contact with friction) as well as nonlinear material, and conducting the time consuming calculations.

The sheets are placed on the horizontal worktable of a guillotine, and next, they are pressed by means of a pressure beam with a force of negligible value. Furthermore, the presence of the pressure beam in the following case is needed to avoid buckling (bulging out) of the metal sheets during cutting. After the metal sheets have been compressed, the main cutting process begins; the cutting tool (knife) moves downwards with uniform rectilinear velocity (*v* = 10 mm/s) until all the sheets have been cut off. Next, it halts on the upper surface of the worktable and comes back to the starting position. In the final step, the pressure beam is liberated, while the material that will be cut is placed in an appropriate position, and the cutting process repeats cyclically.

The model of ultra-thin metal sheets being cut has been discretised into coplanar finite elements responsible for the plane state of strain [45] (Figure 2).

The applied finite element consists of four nodes with two degrees of freedom in a single node that constitute the displacements along x and y axes, respectively. The mesh size was estimated optimally on the basis of the experimental and numerical data, which additionally were matched up with the research results published in scientific papers [17,24] so far. The mesh size of the sheets being cut was established at 0.02 mm in the horizontal direction and 0.01 mm in the vertical direction, respectively. The data concerning the assumed physical model are juxtaposed in Table 2.

It should be highlighted that very fine mesh might lead to the stress singularities, especially at points where the concentrated force has been applied [40,43] e.g., at the tip of a blade. Moreover, further errors might be produced by the computer system during approximation of low floating-point numbers while rounding them. Generally, the smaller the mesh size, the more accurate the results (small errors), but the calculations become more time consuming. So, the equilibrium between the precise results and the time of numerical simulations must be achieved. If the dimensions of the finite elements are large, the time of calculations drops down, but the value of the error level becomes higher. It is normally brought about by the established linear shape function and the numerous nodes.

The ultra-thin metal sheets have been modelled as deformable; therefore, there are many problems encountered during the cutting process mainly connected with such defects as edge bending, vertical scratches, and burrs, and that is why the sheets were subjected to the detailed analysis. Moreover, the sheets are rather flexible on account of their small thickness, which equals 0.1 mm and corresponds to the thickness of an average paper sheet. It should be also stressed that this behaviour is related to the small stiffness of such ultra-thin metal sheets in comparison with other parts of the cutting station. The remained items—i.e., knife, pressure beam, and worktable—are treated as rigid bodies for the sake of their large dimensions in comparison to the thickness of the sheets being cut; for example, the average thickness of the pressure beam is ca. 60 mm and the thickness of the knife is 11 mm. The details concerning the dimensions of a sheet being cut and the cutting tool are juxtaposed in Table 3.

The unilateral constraints (contact with friction) have been imposed between such parts as follows (Figure 2):

- The knife and the top sheet being cut,
- The knife and the bottom sheet being cut,
- The knife and the worktable,
- The pressure beam and the top sheet being cut,
- The top sheet being cut and the bottom sheet being cut,
- The second sheet being cut and the worktable.

In this paper, the contact penalty method [41] for modelling of the contact between the above-mentioned parts in the cutting process has been applied.

The static coefficient of friction is required for calculations involving the exertion of force produced by a pressure beam compressing the bundle of metal sheets, which are mutually acting on one another. Moreover, the bottom sheet executes the pressure on the worktable. These parts are immovable, although the cutting process is associated with motion coming mainly from the rectilinear movement of the cutting tool. The kinetic coefficient of friction is also needed as the cutting tool performs a translational motion. The values of those coefficients for ground and dry surfaces of steel have been taken from the literature [46,47]. The appropriate values for static and kinetic friction coefficients are as follows: *μ_s_* = 0.22 and *μ_d_* = 0.11, respectively, for all parts involved in contact.

The worktable, pressure beam and the part of sheets placed directly under the pressure beam located on the left of the cutting line have been assumed as stationary. The cutting tool and cut off sheets situated on the right side of the cutting line have been assumed as movable.

**Figure 3:**
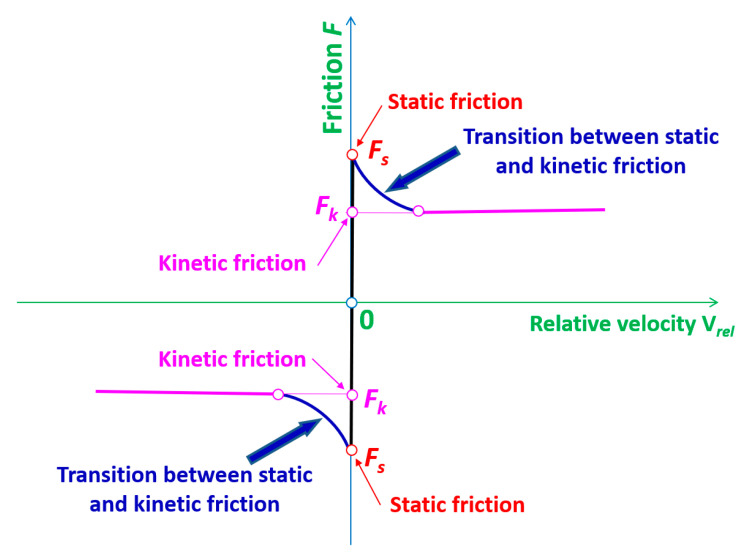
Friction force versus relative velocity.

The effect of relative velocity on the friction value and the interface between both kinds of friction have been considered in the numerical computations (Figure 3). The exponential decay coefficient, *φ* = 500, has been adopted [44], and the Coulomb and Moran’s laws have been applied in the following form [46,47]:

(1) F=μ·N

where *µ*—coefficient of friction and *N*—normal force.

The direction of normal force is perpendicular to the surfaces of sheets being cut. The values of friction force have been estimated for each individual iteration by LS-DYNA software. The friction coefficient (µ) has been established according to Equation (2) [44]:

(2) $$\mu =\mu_{d}+\left(\mu_{s}-\mu_{d}\right)\cdot e^{-\varphi \cdot |V_{rel}|},$$

where *µ_s_*—coefficient of friction in statics, *µ_d_*—coefficient of friction in kinetics, *φ*—coefficient of decay, and *V_rel_*—relative velocity between considered bodies.

## 3. Results and Discussion

For the purposes of this work, the numerical as well as experimental investigations were carried out. The numerical simulations of the cutting process have been conducted by means of a commercial computer system LS-DYNA [44] based on the finite element method and are presented in the form of contour maps of equivalent Huber–Mises stresses and strains corresponding to them. The experimental results have been obtained in the form of images of a blade of a cutting tool and the material being cut, applying scanning electron microscopy. At the end of this chapter, the numerical results have been compared to the experimental data and submitted to verification.

The defects on a surface of sheets being cut are connected with the machining of a cutting tool. Cutting tools are usually ground in the final phase of machining. A grinding machine is one of powerful tools or machine tools used for grinding; it is a type of machining using an abrasive wheel as the cutting tool. Each grain of abrasive on the wheel’s surface cuts a small chip from the workpiece via shear deformation. Grinding machines remove material from the workpiece by abrasion; however, micro grooves remain on the external surface of the cutting tool. Hence, these groves represent the micro defects on a surface of a blade of a cutting tool, which is used as a professional cutting tool in guillotines. Such a knife blade during cutting the metal sheets arranged in a bundle acts with a force high enough to separate metal sheets. The cutting force is so high that the micro defects produced on a knife as a result of its machining are transmitted by mirroring on the cut surfaces of sheets and constitute not only micro imprints but also randomly cause more serious defects in the shape of vertical scratches or craters. The final effect of damage on cut surfaces usually depends on the orientation of the track of grooves resulting from the machining of a knife.

Generally, the defects might be divided into two main categories concerning respectively:

- Defects on a blade of a cutting tool, and
- Defects on a cut surface of a sheet being cut.

The defects on a blade of a cutting tool might form the following:

- Build up edge, and
- Dent in various shapes (e.g., semicircle).

The defects on a surface of a sheet being cut can be divided into the following:

- Edge bending,
- Vertical scratches, and
- Deep vertical grooves or craters.

### 3.1. Numerical Investigations of a Bundle of Metal Sheets Being Cut

The results in the form of equivalent Huber–Mises stresses and corresponding to them equivalent plastic strains for selected time instants have been juxtaposed in order to show the mechanism of the cutting process with usage of the symmetrical blade of the cutting tool. The numerical simulations were carried out for a bundle consisting of two metal sheets. The geometry of a symmetrical blade of the cutting tool with an apex angle *α* = 30° has been applied.

In the numerical simulation of the cutting process, the explicit integration scheme has been utilised. The time step has been controlled by the acoustic wave propagation through the material. It should be emphasised that the numerical stress wave has always propagated less than a single finite element width per time step. The time step of an explicit analysis has been determined as the minimum stable time step in any deformable finite element in the mesh. The applied Courant–Friedrichs–Lewy (CFL) condition has determined the stable time step in each finite element.

For the analysed cutting problem, the actual theoretical time step equals ∆*t* = 1.69·10^−6^ ms, and the time step used during calculations has been self-adjusted and was less than the permissible one (∆*t* = 1.69·10^−6^ ms).

By comparison of the total energy with the external work, it is possible to count the maximum relative error, which equals 0.72% and corresponds to the final time in the numerical analysis. Moreover, the hourglass energy during the numerical calculations of the cutting process does not exceed 0.94% of the total energy. The manual for LS-DYNA [44] states that the results might be acceptable if the hourglass energy is lower than 5% of the total energy. In the considered numerical analysis, the hourglass energy (0.94%) is less than the permissible level (5% of the total energy).

The cutting process of each metal sheet in a bundle runs as follows: in the beginning, the separation of the sheet consists mainly in shearing, and gradually, it is transformed into the totally different mechanism based on ripping resulting from stretching.

**Figure 4:**
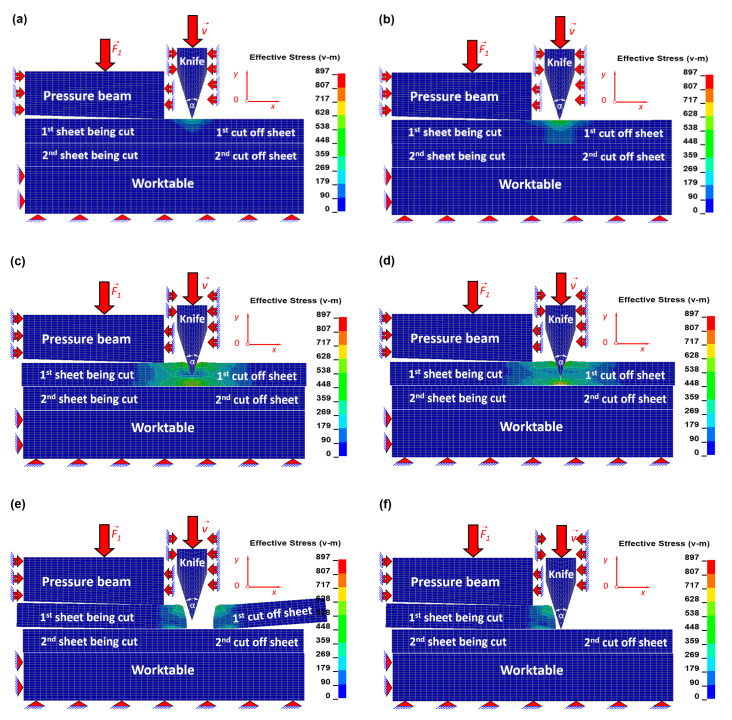
Contour maps of equivalent Huber–Mises stress [MPa] in the top sheet in a bundle being cut for the chosen time instants [ms]: (**a**) *t* = 2.04, (**b**) *t* = 2.07, (**c**) *t* = 8.00, (**d**) *t* = 8.40, (**e**) *t* = 8.43, and (**f**) *t* = 12.00.

**Figure A1:**
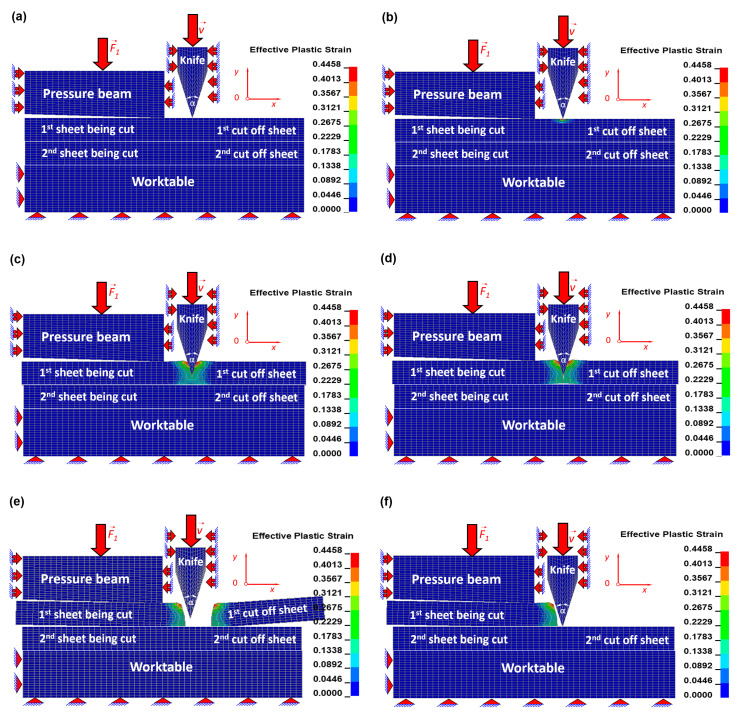
Contour maps of equivalent Huber–Mises plastic strain (mm/mm) in the top sheet in a bundle being cut for the chosen time instants (ms): (**a**) *t* = 2.04, (**b**) *t* = 2.07, (**c**) *t* = 8.00, (**d**) *t* = 8.40, (**e**) *t* = 8.43, (**f**) *t* = 12.00.

In Figure 4, the equivalent Huber–Mises stresses and corresponding to them the equivalent plastic strains (Figure A1 in Appendix A) in the first top metal sheet in a bundle being cut for selected time instants have been presented.

In the beginning of the process, the cutting tool moves downwards, touching the first sheet from the top, and the force required for sheet separation starts to generate. In the very early stage in the material of the sheet being cut, the elastic state of stress and corresponding to it the equivalent elastic state of strain is created within Hook’s law (Figure 4a and Figure A1a). In the next stage, the force generated between the cutting tool and the sheet is so high that it corresponds to the final stage of elastic limit but is still within Hook’s law and simultaneously corresponds to the beginning of nonlinear plastic behaviour (Figure 4b and Figure A1b). It results from exceeding the so-called yield strength (R_e_). In this particular stage, the cutting tool immerses deeper and deeper into the material being cut as a result of shearing. Simultaneously, in the direct vicinity of the blade in the upper part of a top sheet, the compression occurs, while in the lower part of this sheet, the tension appears. During the plastic shearing in metal sheets (Figure 4b,c and Figure A1b,c), many kinds of defects are produced. Edge bending, vertical scratches in the shape of craters, and burrs belong to the most popular defects [2,17,18,19,20,21,22,23,24]. The frequency of their occurrence depends on the sharpness of the blade and quality of external surface of a cutting tool (e.g., lack of built-up edges, dents, etc.) [20,21]. In the final stage of cutting, the shearing mechanism in the upper part of a top sheet ceases to act, and tension in the lower part of this sheet starts to dominate, which in consequence leads to the brittle cracking of the material being cut (Figure 4c,d and Figure A1c,d). It is worth mentioning that the cracking surfaces of a sheet being cut are smooth almost without defects, and that is why the height of the brittle cracking should be maximised. In the final stage of cutting, the first sheet has been separated, and the cut off piece falls away (Figure 4e,f and Figure A1e,f).

The cutting process of the second sheet in a bundle is quite similar to the cutting of the first one; however, there are some differences. The difficulty mainly consists in harder cutting conditions resulting mainly from the plastic deformation of the top sheet, which influences the bottom one—the edge bending produced earlier in the first sheet causes the increased stress in the second one located underneath the first one. Hence, the prestress in the second sheet is higher in comparison to the prestress in the first sheet, and that is why it is easier to cut the second one. The numerical simulations confirm this phenomenon; the second sheet with the prestress on the cutting line has been finally cut off ca. 5 ms faster in comparison with the time of cutting of the first one (without prestress on the cutting line).

**Figure 5:**
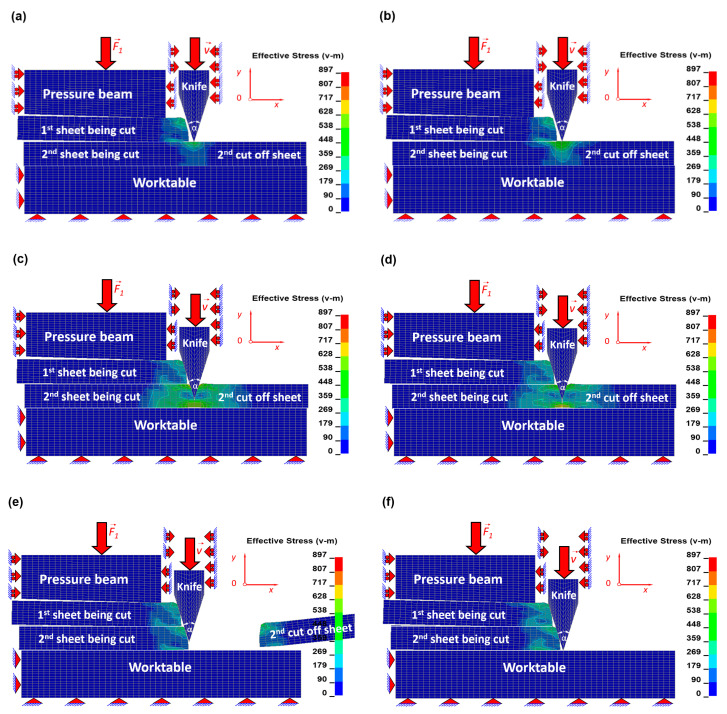
Contour maps of equivalent Huber–Mises stress [MPa] in the bottom sheet in a bundle being cut for the chosen time instants [ms]: (**a**) *t* = 12.04, (**b**) *t* = 12.07, (**c**) *t* = 18.00, (**d**) *t* = 18.35, (**e**) *t* = 18.43, (**f**) *t* = 22.00.

**Figure A2:**
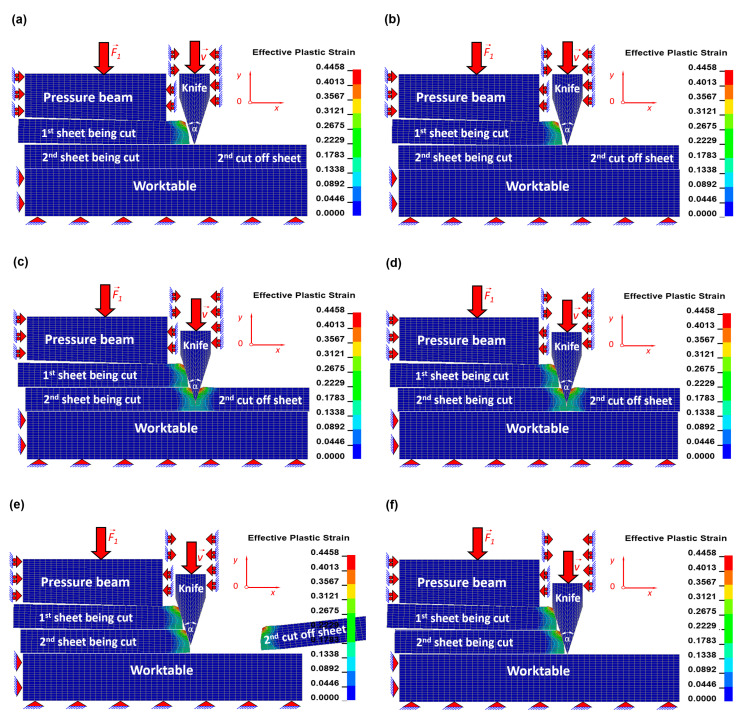
Contour maps of equivalent Huber–Mises plastic strain (mm/mm) in the bottom sheet in a bundle being cut for the chosen time instants (ms): (**a**) *t* = 12.04, (**b**) *t* = 12.07, (**c**) *t* = 18.00, (**d**) *t* = 18.35, (**e**) *t* = 18.43, (**f**) *t* = 22.00.

In the first stage, during cutting of the second sheet, similarly to cutting the first sheet in a bundle, the elastic zone is created (Figure 5a and Figure A2a). Immediately after it, the plastic shearing (Figure 5b,c and Figure A2b,c) responsible for the formation of numerous defects occurs, and finally, the brittle cracking (almost without apparent defects) begins to dominate, which has been presented in Figure 5c,d and Figure A2c,d. In the final stage of cutting, the second sheet has been separated, as it was mentioned before about 5 ms earlier, and the cut off piece falls away (Figure 5e,f and Figure A2e,f).

It should be noted that the highest value of stresses in the vicinity of the cutting line is slightly bigger in comparison to the cutting of the first sheet, and furthermore, the total period of time destined for cutting is slightly shorter than in the case of the first sheet. Before the fracture occurs in the cut off sheet, the elastic as well as the plastic energy has been accumulated, and directly after the sheet has been separated, the elastic energy is instantly released, causing the cut off pieces of the sheets to move. It is also worth noting that the total energy stored in the second sheet is slightly higher than that in the first one.

The numerical calculations as well as experimental investigations evidently show that the first sheet being cut (the top one) undergoes the easiest cutting conditions in comparison to the other one located underneath in a bundle.

By harder cutting conditions, it should be understood that the remaining particles after cutting of the top sheet (which was separated as a result of plastic and brittle cracking) are taken by a blade of the cutting tool and form a random micro defect on it before cutting the next sheet. It should be stressed that the brittle cracking happens before the knife travelled the whole distance corresponding to the thickness of a single sheet (Figure 4e and Figure 5e, Figure A1e and Figure A2e). The next sheet in a bundle is cut with the micro defect already randomly formed on the blade, which causes macro defects on the bottom sheet in a bundle. The defects occurring on the surfaces of sheets being cut are more numerous in the bottom sheet.

The easiest cutting conditions are understood as the smallest possible deflection of the edge bending, the minimum height of the plastic region caused by shearing in the early stage of cutting process succeeding immediately after the elastic deformation (of the edge bending), and the maximum height of the brittle cutting zone caused by cracking resulting from too high tensile strength in the final stage of the cutting process. The cracking surface of the sheet being cut is quite smooth, without vertical scratches, without burrs, and edge bending, and that is why it is highly desired. The future scientific investigations should be focussed on maximisation of this characteristic brittle zone with simultaneous reduction of the plastic zone responsible for numerous defects such as:

- Deflection of the edge bending of the sheet being cut,
- Random occurrence of deep vertical scratches in the shape of craters, and
- Random occurrence of burrs.

The inertia effect has been taken into consideration mainly in numerical calculations. It can be easily observed immediately after each metal sheet has been cut off. The inertia effects accompany the sheets being cut during the entire cutting process, which consists of the three following phases: cutting until the sheet is not thoroughly separated, the moment of complete separation (due to fracture), motion of the sheet after its separation.

### 3.2. The Scanning Microscope Observation of a Symmetrical Cutting Tool

The experimental investigations of a cutting tool and sheets being cut have been carried out using a scanning electron microscope with the cold field emission (FESEM) HITACHI S-4700 (Hitachi, Ltd., Tokyo, Japan) equipped with an energy dispersive X-ray (EDS) NORAN Vantage spectrometer (Noran Co, Vernon, CA, USA).

**Figure 6:**
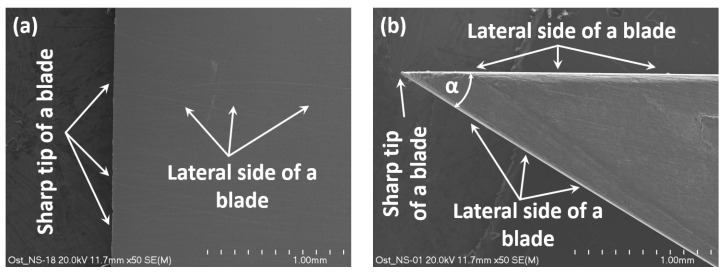
The blade of a cutting tool under magnification ×50; view from the lateral side (**a**) and from the front (**b**).

The symmetrical cutting tool was made of steel C75S in the shape of an isosceles triangle with an apex angle *α* = 30° (Figure 2 and Figure 6). The cutting tool used in the experiment was brand new; under magnification ×50, the tip of its blade looks sharp. The external surfaces of the blade of a cutting tool are quite smooth without apparent scratches coming from machining, which implies that the quality of the knife is high.

### 3.3. The Scanning Microscope Observation of Surfaces of Sheets Being Cut

**Figure 7:**
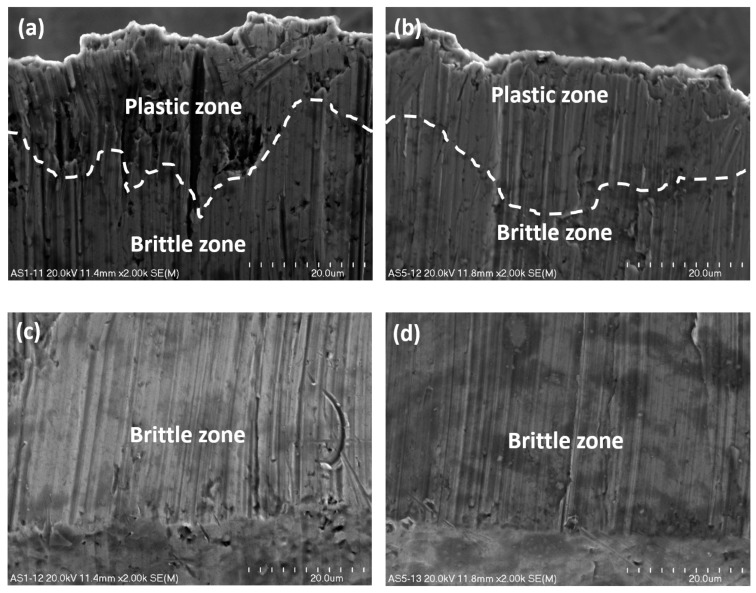
The upper part (**a**,**b**) and the lower part (**c**,**d**) of a surface of the top sheet in a bundle zoomed in ×2000; view from the front (**a**,**c**) and from the rear of a knife (**b**,**d**).

**Figure 8:**
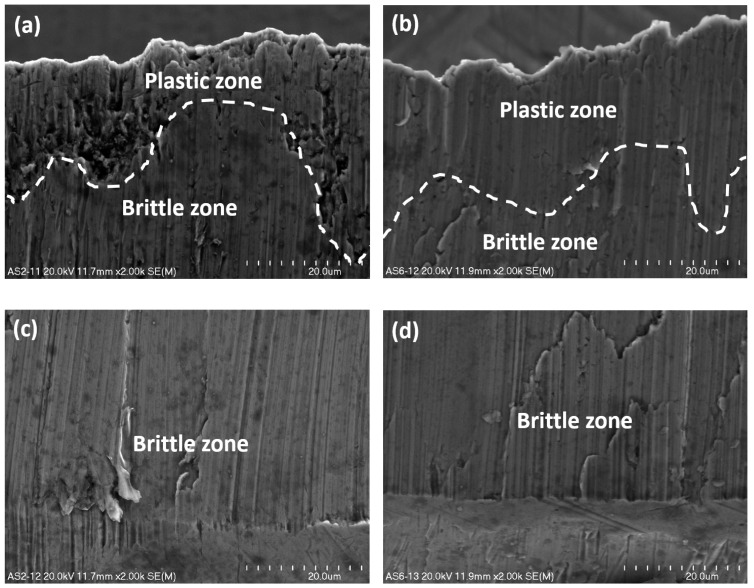
The upper part (**a**,**b**) and the lower part (**c**,**d**) of a surface of the bottom sheet in a bundle zoomed in ×2000; view from the front (**a**,**c**) and from the rear of a knife (**b**,**d**).

The material being cut was prepared in the form of a bundle comprising two ultra-thin cold-rolled steel sheets (C75S), and next, it has been cut by a knife presented in the previous subsection (Figure 6). The surfaces of metal sheets that were cut off have also been observed under scanning electron microscope under magnification ×2000. The images of the top sheet in a bundle are juxtaposed in Figure 7, and the images of the bottom sheet are juxtaposed in Figure 8.

The front side and the back side of a cutting tool differs to a great extent with reference to the essence of the fracture. The textures of a top steel sheet in a bundle being cut viewed from the front of a knife are presented in Figure 7a,c, and the morphological features of cut surfaces viewed from the rear of a knife are presented in Figure 7b,d.

In the upper part of the first sheet being cut in a bundle, there is a characteristic plastic zone resulting mainly from shearing. Underneath the plastic zone, a brittle zone starts forming as a result of the cracking process coming from the tensile tension (Figure 7a,b). In the lower part of the earlier mentioned sheet, only the brittle zone is observed (Figure 7c,d). In the plastic zone, numerous defects in the shape of spherical and ellipsoidal micro-voids of various sizes are visible. Their formation is caused by the arising nucleating sites, and because they are situated in close distances (Figure 7a), they often merge with one another before growing to bigger dimensions [48]. The brittle walls were watched in the lower part of the sheet being cut, but micro-voids were not detected.

The second sheet being cut behaves similarly to the first one in the sense that there are two characteristic zones: plastic and brittle (Figure 8). The main distinction consists in the size and number of defects in the shape of micro-voids observed in the plastic zone; they are slightly larger and more numerous (Figure 8a). In the lower part of the bottom sheet, a larger number of brittle walls arise (Figure 8c,d) than in the case of the first sheet being cut.

The structure of cross-section of sheets being cut, taking into account the plastic zone, depends on the position of the knife. If the process is watched from the front of the knife, the cut surfaces are non-homogenous (Figure 7a and Figure 8a), otherwise, when the investigation is conducted from the rear of the knife; they are homogenous (Figure 7b and Figure 8b). In terms of the brittle zone, the cross-section of both sheets is rather smooth and homogenous (Figure 7c,d and Figure 8c,d).

The area of the cut surfaces of the steel sheet which corresponds to approximately 1/5 of its height measured from the top downward is submitted to plastic fracture, and the other part of the cut surface equal to approximately 4/5 of the sheet height measured from the bottom upward is submitted to brittle fracture. The characteristic interfacial passage between the plastic and brittle area has been designated by a white dashed line and is presented in Figure 7a,b as well as in Figure 8a,b. It is evident that the interfacial transition is rather nonlinear and varies along the whole width of the cross-section after the sheet has been cut off.

Comparing the ductile zone in both sheets viewed from the front and back of the cutting tool, it can be stated that both are deformed plastically with many various defects due to shearing, although those regions located in the front of the knife are more corrupted (Figure 7a and Figure 8a). On these surfaces, many dimples characteristic for plastic damages are visible, while in those surfaces seen from the rear of the knife, the dimples have not been detected (Figure 7b and Figure 8b).

Comparing the brittle zone viewed from the front (Figure 7c and Figure 8c) and back (Figure 7d and Figure 8d) of the cutting tool, it can be affirmed that in both sheets, the surfaces are flat and smooth practically without defects; however, there are some minor differences in terms of the top and bottom sheet in a bundle. Taking into account the first sheet, there are no evident detected differences between the front (Figure 7c) and the back of the knife (Figure 7d). Considering the second sheet, the surfaces viewed from the front of the cutting tool are less defected because a smaller number of brittle walls is present (Figure 8c) with the respect to the surfaces viewed from the rear of the cutting tool (Figure 8d).

In the present paper, only two sheets have been considered using a symmetrical knife. The morphological features of the cross-sections are similar to those investigated by Kaczmarczyk et al. [17] concerning the cutting of a bundle comprising two sheets with a non-symmetrical knife. The size of the plastic and brittle fracture constitutes the main distinction; in the case of a symmetrical blade of a knife, the size of the plastic area is substantially smaller than in the variant of the cutting process by means of a non-symmetrical knife.

### 3.4. Comparison of the Numerical and Experimental Investigations

**Figure 9:**
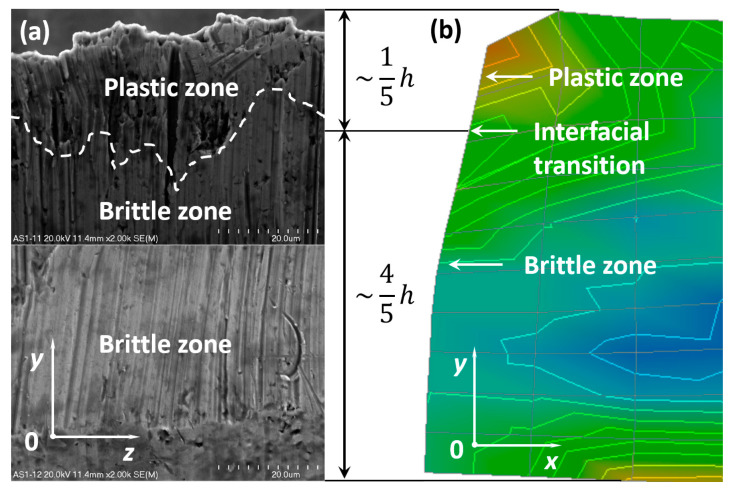
Comparison of the experimental (**a**) and numerical results (**b**) for the top sheet in a bundle.

The numerical results in the form of contour maps representing the equivalent Huber–Mises stresses for the time instant immediately after the metal sheet has been cut off (Figure 9b) have been compared with the scanning electron microscopy images under magnification ×2000 (Figure 9a). The comparison evidently shows that at the upper part of the top metal sheet being cut, the plastic region is formed. For the applied symmetrical cutting tool, the height of the plastic zone measured from the top of a sheet downwards is ca. 1/5 of the total height of the whole sheet (*h* = 0.1 mm). Underneath the plastic zone, a brittle one has been observed. The height of the brittle zone measured from the bottom of a sheet upwards equals ca. 4/5 of the total height of the sheet.

**Figure 10:**
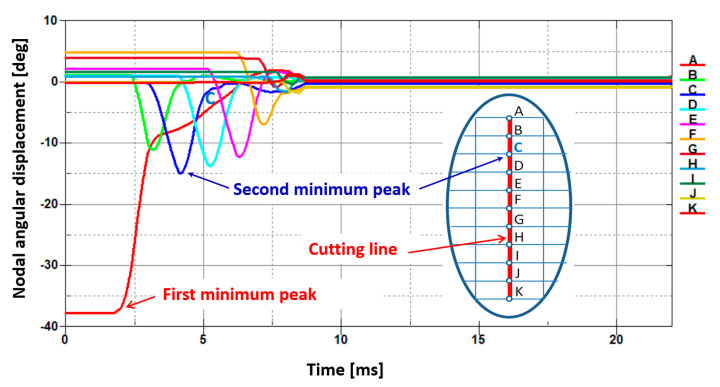
Juxtaposition of changes of an angular displacement in the function of time for each node situated on the cutting line and denoted by letters from A to K for the top sheet in a bundle with respect to the model presented in Figure 2.

In order to prove numerically that the interfacial transition line between the plastic region and the brittle one occurs, the appropriate characteristics of angular displacements have been performed and presented in the form of graphs (Figure 10). These characteristics represent changes of nodal angular displacements versus time for all nodes belonging to the cutting line from A to K for the top sheet in a bundle. The first minimum peak corresponds to the first node marked by letter A presented previously in Figure 2 in detail. It seems to be quite obvious that in this particular node, the angular displacement attains the minimum value because it is the first node in which the elastic and then the plastic shearing occurs. It is worth mentioning that for the second minimum peak, it is not so apparent. It has been found that the second minimum peak corresponds to the transition line between the plastic and brittle zone after many efforts concerning the research devoted to different geometry of a cutting tool [24]. The second peak corresponds to the node C lying on the cutting line. Thus, this interfacial transition has been determined on the basis of numerical calculations (Figure 9b) and is in quite good agreement with the results of experimental investigations (marked by white dashed line in Figure 9a). Recapitulating, above node C, the plastic region occurs and below node C, the brittle one appears. Hence, node C belonging to the transition line separates the plastic and brittle zone. This characteristic location of node C confirms also the experimentally found transition line marked by a white dashed line in Figure 9a.

**Figure 11:**
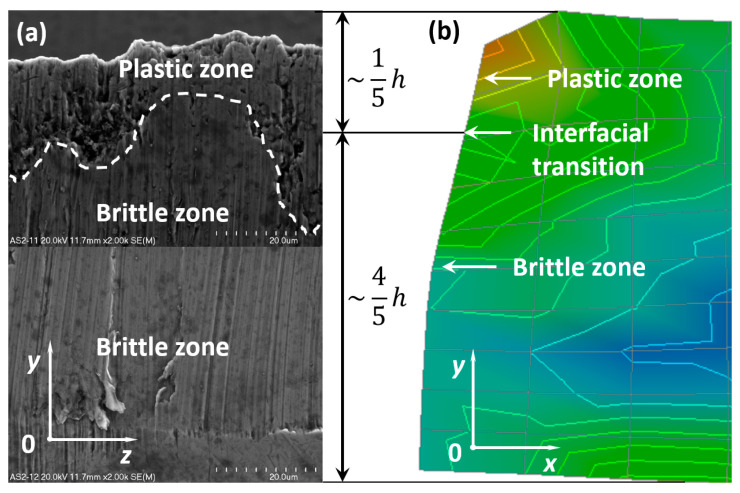
Comparison of the experimental (**a**) and numerical results (**b**) for the bottom sheet in a bundle.

**Figure 12:**
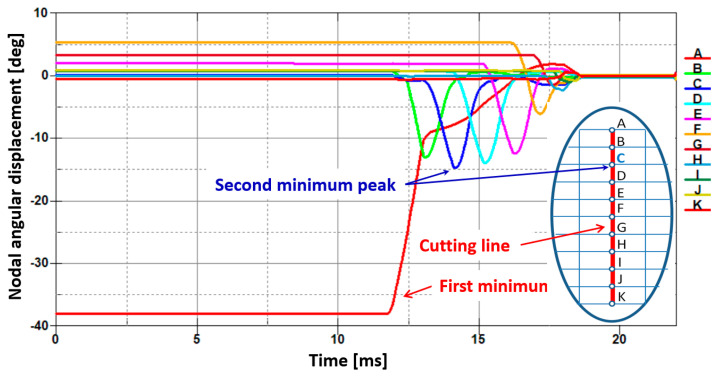
Juxtaposition of changes of an angular displacement in the function of time for each node situated on the cutting line and denoted by letters from A to K for the bottom sheet in a bundle with respect to the model presented in Figure 2.

The second sheet being cut behaves similarly to the first one in the sense that there are two characteristic zones: plastic and brittle (Figure 11). Similarly to the first sheet, the relationship between the change of an angular displacement for all nodes along the cutting line versus time has been presented in the form of graphs (Figure 12). The curve corresponding to the second minimum peak represents the boundary line between the plastic and brittle regions. The graphs in Figure 12 are shifted in time with respect to the graphs presented in Figure 10.

The main distinction between both sheets consists in the size and number of defects in the shape of micro-voids observed in the plastic zone; they are slightly larger and more numerous in the bottom sheet. In the lower part of the bottom sheet, a larger number of brittle walls arose than in the first sheet.

For both sheets, the transition line can be approximately identified as a node located on the cutting line and corresponding to the point C shown in Figure 2. In this particular node, the angular displacement reaches its second minimum (Figure 10 and Figure 12). It should be also highlighted that the first minimum of an angular displacement corresponds to the first node lying on the cutting line (point A) for both sheets in a bundle. In this peculiar node located at the top of the cutting line, the shearing stress dominates and causes the highest plastic deformation. The transition line between these two characteristic zones found numerically allows engineers to identify the plastic zone that is located above point C and the brittle zone situated below point C. Thus, point C corresponds approximately to the mentioned transition between both characteristic regions for both sheets in a bundle.

The most important question comes to mind: “Is it possible to propose such geometry of the blade of a cutting tool for which the height of a plastic zone can significantly reduced?” The author of this article is aware of the difficulties connected with this kind of innovative solution.

The earlier scientific investigations [17] show that the plastic zone is much larger for a non-symmetrical cutting tool, which equals circa 1/3 of the height of the whole sheet compared to the height of the plastic zone, which approximately equals 1/5 of the total height of a sheet for a symmetrical cutting tool. It evidently proves that the height of the plastic zone depends strongly on the geometry of the cutting tool. It should be also noted that the nose angle of the blade of a cutting tool was the same (*α* = 30°), and moreover, the material being cut as well as the cutting conditions were almost the same. It confirms that the height of an advantageous brittle zone can be maximised simultaneously with reduction of the height of an undesired plastic zone.

In the cutting process, one may mentally distinguish two zones that are responsible for fracture types I and II connected with the residual stresses. Similar issues were investigated by E. Salvati and A.M. Korsunsky and concerned the presence and nature of inter- and intra-granular residual stresses (type II and III) that were present in an aluminium alloy sample as a consequence of plastic deformation [49].

Recapitulating, the plastic and brittle zone as well as the transition between these regions can be found experimentally as well as numerically. The comparison is presented in Figure 9 and Figure 11.

## 4. Conclusions

In this paper, the cutting process of a bundle of ultra-thin cold-rolled steel sheets (C75S) has been taken into consideration. To show the mechanism of cutting of sheets arranged in a bundle, the author’s physical models and corresponding to them the mathematical ones have been elaborated by means of the finite element method. The numerical time-consuming dynamical calculations have been performed applying the computer system LS-DYNA. It should be noted that the computations are highly nonlinear; therefore, the material and geometrical nonlinearities understood as unilateral constraints imposed on all nodes undergo so-called contact with friction. The experimental investigations consisted in observation of the surfaces of sheets submitted to cutting under large magnification (×2000). Finally, the nonlinear dynamical fast changing numerical results have been compared with experimental data, and on this basis, the following conclusions have been drawn:

- The first sheet in a bundle can be quite easily cut off, but the conditions for the sheet located underneath in a bundle are slightly harder, because the plastic deformation arisen in the first sheet influences the second one (located on the bottom of a bundle),
- The plastic zone of sheets being cut equals circa 1/5 of the total height of a sheet, but the plastic zone for the second sheet (located on the bottom in a bundle) is connected with a slightly higher number of defects caused by higher shearing stresses, which means that the cutting conditions might be determined as harder,
- The brittle zone of sheets being cut equals circa 4/5 of the total height of a sheet, but the brittle zone for the second sheet (located on the bottom) is connected with a slightly higher number of brittle walls with no micro-voids caused by a higher equivalent Huber–Mises stresses, which means that the cutting conditions can be described as harder, too,
- The high values of equivalent Huber–Mises stresses and corresponding to them strains are in sheets situated on the cutting line directly underneath the tip of a blade,
- The stresses and strains located along the cutting line are slightly higher in the bottom sheet in comparison to the top one,
- The failure mechanisms concerning the surfaces of sheets being cut exerts influence on:
  - Shearing stresses, which are responsible for causing undesired various damages especially in the plastic region such as dimples, micro voids, scratches, etc., in the early phase of the cutting process,
  - Tensile stresses, which are responsible for attaining smooth surfaces practically without defects except those observed in shape of numerous brittle walls due to ripping of sheets being cut into separate parts in the final phase of the cutting process,
- The size of the plastic region responsible for most damages is strongly dependent on the geometry of the blade of a cutting tool. It is possible to influence the reduction of undesired plastic region as well as maximisation of the highly desired brittle one by changing the geometry of the blade of a cutting tool.

## Figures and Tables

**Table 1:** Mechanical properties of material being cut—cold-rolled streel C75S.

No	Name of the Material Properties	Symbol	Value [17,23,46]
1.	Young’s modulus	*E*	205 GPa
2.	Poisson’s ratio	*ν*	0.28
3.	Kirchhoff’s modulus	*G*	80 GPa
4.	Tangent modulus	*E_T_*	0.867 GPa
5.	Failure strain	*ε_f_*	0.15 mm/mm
6.	Yield strength	*R_e_*	0.51 GPa
7.	Ultimate tensile strength	*R_u_*	0.64 GPa

**Table 2:** Details concerning parts discretisation.

No	Name of the Part	Kind of Part	Number of Nodes	Number of Finite Elements
1.	Cutting tool	Rigid	208	180
2.	First metal sheet in a bundle	Deformable	572	500
3.	Second metal sheet in a bundle	Deformable	572	500
4.	Pressure beam	Rigid	546	500
5.	Immovable worktable	Rigid	1071	1000
Total number in whole model			2969	2680

**Table 3:** Dimensions of a cutting tool and sheet being cut.

No	Dimension/Part	Length [mm]	Width [mm]	Thickness [mm]
1.	Cutting tool	30	35	11
2.	Sheet being cut	2.0	12.5	0.1

## Appendix A

The equivalent Huber–Mises plastic strains [mm/mm] in the top metal sheet in a bundle being cut for the selected time instants have been presented in Figure A1.

The equivalent Huber–Mises plastic strains (mm/mm) in the bottom metal sheet in a bundle being cut for the selected time instants are presented in Figure A2.
